# Early Steps of Mammary Stem Cell Transformation by Exogenous Signals; Effects of Bisphenol Endocrine Disrupting Chemicals and Bone Morphogenetic Proteins

**DOI:** 10.3390/cancers11091351

**Published:** 2019-09-12

**Authors:** Nora Jung, Veronique Maguer-Satta, Boris Guyot

**Affiliations:** 1 CNRS UMR5286, Centre de Recherche en Cancérologie de Lyon, F-69000 Lyon, France; nora.jung@lyon.unicancer.fr; 2 Inserm U1052, Centre de Recherche en Cancérologie de Lyon, F-69000 Lyon, France; 3 Université de Lyon, F-69000 Lyon, France; 4 Department of Tumor Escape Signaling, Centre de Recherche en Cancérologie de Lyon, F-69000 Lyon, France; 5 Institut des Sciences Pharmaceutiques et Biologiques, Université Lyon 1, F-69000 Lyon, France

**Keywords:** bone morphogenetic protein (BMP), epithelial stem cells, breast cancer, bisphenol, estrogens, microenvironment

## Abstract

Estrogens are major regulators of the mammary gland development, notably during puberty, via estrogen receptor (ER) activation, leading to the proliferation and differentiation of mammary cells. In addition to estrogens, the bone morphogenetic proteins (BMPs) family is involved in breast stem cell/progenitor commitment. However, these two pathways that synergistically contribute to the biology of the normal mammary gland have also been described to initiate and/or promote breast cancer development. In addition to intrinsic events, lifestyle habits and exposure to environmental cues are key risk factors for cancer in general, and especially for breast cancer. In the latter case, bisphenol A (BPA), an estrogen-mimetic compound, is a critical pollutant both in terms of the quantities released in our environment and of its known and speculated effects on mammary gland biology. In this review, we summarize the current knowledge on the actions of BMPs and estrogens in both normal mammary gland development and breast cancer initiation, dissemination, and resistance to treatment, focusing on the dysregulations of these processes by BPA but also by other bisphenols, including BPS and BPF, initially considered as safer alternatives to BPA.

## 1. Introduction

Breast cancer is the most common cancer in women and exhibits important phenotypic and genetic diversities associated with different prognostics. Breast cancers are clinically classified based on histological appearance and expression of hormone receptors such as estrogen (ER) and progesterone (PR) receptors, as well as on the amplification of the Her2 gene coding for a member of the EGF receptor family [1]. Based on these criteria, four major breast cancer subtypes have been defined: Luminal A and luminal B (all ER+), HER+ (that can be either ER− or ER+), and basal-like, triple negative (as such ER−) tumors [2,3]. The ER status in breast tumors is determined by immunohistochemistry detection of the nuclear expression of the classical 66 kDa isoform of ERα (ERα66). In ER-positive tumors, preventing ER activation is an efficient therapy. This can be achieved either by using competitive antagonists of estrogens (e.g., Tamoxifen), preventing its binding to and subsequent activation of ER, by using drugs blocking estrogen synthesis (anti-aromatase) in post-menopausal women, or by luteinizing hormone-releasing hormone (LHRH) analogs, inhibiting female hormones release by the ovaries [4].

Based on epidemiological studies, different factors increasing the risk of breast cancer development have been highlighted. These factors can be intrinsic, such as mutations in Brca1 or 2, Tp53 or ATM, or extrinsic, e.g., related to the environment or lifestyle [5,6]. While different genetic alterations appear progressively following different oncogenic signals, hereditary mutations in breast cancer-predisposing genes likely account for approximately 10% of breast cancers [7,8]. In breast cancer with a genetic origin, the most commonly mutated genes are Brca1 and Brca2 [7]. BRCA1 and 2 are two major regulators of DNA double-strand break (DSB) repair through homologous recombination (HR) and play a crucial role as tumor suppressor genes, likely by preventing mutations and genome instability [9]. Breast cancer is a multifactorial disease and evidence for the involvement of extrinsic factors in breast cancer risk has been described. Indeed, a lack of physical activity, tobacco or alcohol consumption and contraceptive pills or hormone replacement therapy for post-menopausal women were shown to increase breast cancer risk [10]. As mentioned previously, estrogens are involved in the proliferation of normal mammary cells but also of breast tumor cells through ER stimulation, leading to the activation of several pathways involved in cell proliferation and resistance to apoptosis [11,12]. Hormonal status has been described to play a major role in breast cancer risk, as a premature or extensive exposure to endogenous estrogens (due to an early menarche, nulliparity, late age for first full-term pregnancy or a late menopause) increases the risk of breast cancer [13].

The mammary gland is not only exposed to endogenous hormones but also to endocrine-disrupting chemicals (EDCs), molecules present in the environment able to mimic these hormones. The interest in EDCs is growing rapidly, owing notably to their extensive use in manufactured goods and their release in our environment. Several EDCs involved in breast cancer risk have been identified, including organochlorine pesticides like DDT or DDE, dioxins or polychlorinated biphenyls. In addition, the bisphenol A (BPA) EDC has raised increasing concerns during the past few years due to its widespread presence in our environment [14,15]. BPA is an aromatic compound used by the plastic industry as a monomer in the synthesis of polycarbonates and epoxy resins. Polycarbonates are found in consumer plastic-like water bottles, sport equipment or toys. Epoxy resins are used to coat the inside of food or beverage containers. BPA can also be found in thermal paper. BPA monomers from these compounds can be released into the environment by hydrolysis. Despite rising concerns about its safety [16] and progressive restrictions on its use, several million tons of BPA are still produced worldwide. Due to its estrogen-mimetic properties allowing ER activation [17,18], the role of BPA in breast cancer is of particular interest. Importantly, tumor development is a long and complex process, which includes several steps following transformation initiation by genetic and epigenetic modifications in normal cells. It is thus highly probable that mutations or abnormalities accumulate in a given epithelial cell, ultimately leading to its transformation. This process is accompanied by morphological changes and modifications of key cellular functions (proliferation, survival, and differentiation) until advanced stages of cancer initiation, following which altered cells expand and completely invade the lumen. In this context, these events will preferentially take place in a long-lasting cell of the mammary gland. Indeed, previous data suggest that target cells of the transformation process could be mammary epithelial stem-like cells as documented in the case of the claudin-low breast cancer subtype [19]. In that context, studying the role of BPA on mammary stem cell biology appears to be highly pertinent.

## 2. The Role of Estrogens in Mammary Gland Development and Homeostasis

### 2.1. Production and Function in Early Mammary Gland Development 

The mammary gland begins its development during embryogenesis and is composed of a rudimentary ductal system blocked until puberty. Then, two master reproductive hormones are secreted, namely, estrogens and progesterone. Estrogens control the growth of ducts from their distal extremity called terminal end buds (TEBs) [20,21,22], while progesterone is involved in lateral branch development [23,24].

One of the major hormones involved in mammary gland development is estrogen, mostly produced by the ovaries but also by other tissues including breast, adipose tissue, liver, pancreas, bone, skin, brain, and placenta. Estrone (E1), estradiol (E2), estriol (E3) and estetrol (E4, only produced by the foetus but passing in the mother’s blood) are the four major endogenous estrogens in women. Depending on the life stage of women, these various types of estrogens are differentially expressed, with a predominance for estriol and estetrol during pregnancy, for estrone during menopause, and for estradiol in non-pregnant women between menarche and menopause [25]. Even though a non-aromatase pathway exists [26], the major estradiol biosynthesis pathway involves aromatase enzymes, catalyzing the conversion of androstenedione to estrone that is then converted to estradiol or catalyzing the direct conversion of testosterone into estradiol [27]. 

The effects of estrogens appear at the time of breast development following the establishment of the hypothalamo–hypophyseal axis. During puberty, the hypothalamus releases gonadotropin-releasing hormone (GRH), leading to the secretion of several hormones by the pituitary gland, such as gonadotropins, follicle-stimulating hormone (FSH), and growth hormone (GH) [28]. These hormones drive estrogen production by the ovaries. In addition, it has been shown in the rat mammary gland that GH can directly increase estrogen receptor alpha (ERα) expression in breast stromal cells that surround mammary epithelial cells [29]. Altogether, estrogens, in combination with other hormones, orchestrate the growth of the ductal system and adipose tissue accumulation during puberty [20,21,22].

### 2.2. Importance in the Adult Mammary Gland

The adult mammary gland is formed of ducts and lobules of secreting luminal epithelial cells surrounded by contractile myoepithelial cells. These epithelial cells are embedded in a stroma mainly formed of fibroblasts and adipocytes that secrete several soluble molecules regulating epithelial cell functions and differentiation. Epithelial cells of the mammary gland are generated by mammary stem cells, while the stromal compartment arises from mesenchymal stem cells. During adulthood, the mammary gland undergoes functional and structural changes that alternate between phases of proliferation, differentiation and apoptosis. This is controlled by cyclic hormonal variation due to the estrous/menstrual cycle. However, this post-pubertal mammary tree is still immature and only achieves full maturation during pregnancy and lactation. These final steps encompass alveogenesis and milk production, which take place mostly under the control of progesterone and prolactin [30,31]. Mallepell et al. suggested that estrogens do not directly stimulate proliferation of the ER-positive luminal cells but act via a paracrine process [11,32]. Indeed, ER-positive and proliferative cells were shown to be distinct [33,34]. Estrogens act on luminal ER/PR-positive cells, leading to the cleavage and liberation of amphiregulin [35,36], which affects neighboring ER/PR-negative cells. In addition, these ER/PR-negative cells have characteristics of stem cells (SCs), in which asymmetric division is controlled by growth factors like amphiregulin released by stromal cells [37,38,39,40]. Moreover, when mice are ovariectomized or treated with letrozole (to inhibit endogenous estrogen biosynthesis and provide a normal stromal and hormonal environment for all other hormones), a decrease in the absolute number of repopulating cells and in ductal growth and expansion was reported in the fat pads following transplantation of a limited number of mammary stem cells (MaSCs) [41]. In this setting, an increase in the percentage of MaSCs-enriched cells in the G0/G1 phases, a decrease in the Ccnd1 and Ccdn2 level in MaSCs-enriched population, and a decrease in the size of the luminal cell population were also observed. However, estrogen depletion does not directly impact the size of the MaSCs-enriched subpopulation [41]. 

These data support the hypothesis that ductal elongation controlled by estrogens is dependent on the presence of luminal ER/PR-positive cells which act as estrogens sensors and release in response factors resulting in ER/PR-negative stem cell division. This highlights the importance of the estrogen pathway on MaSC regulation and their potential resultant sensitivity to estrogen-mimetics like BPA.

### 2.3. Effects of EDCs with Estrogenic Activity

Some chemical pollutants have been classified as EDCs based on the following definition: “*an exogenous chemical, or mixture of chemicals, that interferes with any aspect of hormone action*” [15,42]. Estrogens are one of the main hormones altered by EDCs. Perturbations of estrogen functions have been reported in a wide spectrum of pathologies, including metabolic, bone, and reproductive disorders as well as breast, endometrial or ovarian cancers [16]. 

At the cellular level, stem cells are a unique category of cells active from embryogenesis up to late stages of human adult life. Stem cells are fundamental in the development of all organs, in tissue homeostasis and in diseases. Their unique properties, such as an extended life and low cycling features, make these cells privileged targets of long-term exposure to numerous factors and alterations. As important actors of organ integrity, tissue homeostasis and repair, stem cells are likely involved at one step or another in dysregulations of physiological processes that can occur at any stage of life. 

Normal stem cells functions are thus likely to be affected upon exposure to EDCs [43,44,45,46,47]. It has been shown that this exposure occurs throughout life and even during embryogenesis, at the moment of mammary gland establishment. For instance, bisphenol A (BPA) has been detected in urinary samples but also in maternal and fetal plasma, in colostrum and in placental tissue at birth [48]. Several studies have demonstrated that a prenatal exposure to BPA induces changes in fetal mouse mammary glands, in the epithelial compartment as well as the stromal compartment, favoring the fat pad maturation and increasing the mammary gland susceptibility to oncogenes [49,50,51]. Wadia et al. have described transcriptome modifications, notably an increased expression of genes involved in apoptosis prevention, myoepithelial differentiation or adipogenesis and a decreased expression of those involved in cell adhesion [51]. These data highlight an impact on mammary gland development by EDCs, such as BPA.

## 3. Mammary Gland Regulation by the BMP Pathway

One of the major conserved signaling pathways involved in stem cell regulation from embryogenesis up to adult stages is the bone morphogenetic protein (BMP) signaling. There are 21 different soluble BMP molecules that act through serine/threonine kinase BMP receptors (BMPR). In the context of SC regulation, BMP2 and BMP4 progressively emerge as the most important BMPs. Likely through its involvement in SC regulation, the BMP pathway is involved in numerous physiological and pathological processes [52], particularly in tissue regulation. Indeed, BMPs were first described as regulators of skeletal development, and are actually found in the microenvironment of several tissues where they contribute to tissue morphogenesis and homeostasis, such as the mammary gland. It is well known that members of the TGFβ family, which includes BMPs, are involved in different aspects of female reproduction [53,54,55,56]. BMPs were initially identified as key regulators of bone morphogenesis through their function in mesenchymal stem cells (MSCs) regulation, such as lineage specification of adipocytes that are one of the major elements of the mammary gland microenvironment [57,58,59]. Alterations in BMP signaling have been implicated in metabolic disorders such as obesity in women [60,61]. Altogether, it indicates a major importance of BMP signaling in the regulation of different aspects of the mammary gland biology.

### 3.1. Role of BMPs in Mammary Gland Development

The BMP signaling pathway is a dynamic and complex pathway, leading to the transduction of various signals depending on the nature of the BMP ligand and of the BMPR complex oligomerization induced (for review: [62,63]). It has been reported that BMPs may interact with their receptors in two different ways [64,65]: On the one hand, BMPs induce a BMPR complex formation called BISC (BMP-induced signaling complex) and on the other hand, a preformed BMPR complex is present before BMPs fixation, known as PFC (pre-formed complex). These two different modes of BMP signal initiation lead to two different signaling cascades, namely, the canonical SMAD-dependent pathway and the non-canonical SMAD-independent pathway [65]. The SMAD-dependent pathway includes the recruitment of SMAD1-5-8 proteins, leading to their phosphorylation by type 1 BMPR. SMAD-phosphorylated proteins then form a complex with SMAD4, leading to its translocation to the nucleus where it acts as a transcription factor on target genes [62,66]. The SMAD-independent pathway does not simply encompass one signaling pathway but a multitude of downstream cascades, involving p38, Ras/ERK and PI3K/AKT [67,68,69,70]. BMPs are involved in the different steps of the mammary gland development. During embryogenesis in mice, BMP4 was shown to participate in the early steps of mammary gland development by regulating the dorso–ventral axis establishment [71]. The BMP pathway also plays a role in mammary bud formation and outgrowth, as well as in ductal branching morphogenesis initiation. Hens et al. showed that BMP4 is expressed in both mesenchymal and epithelial cells of the mammary bud and that the use of a BMP4 inhibitor leads to a decrease in bud outgrowth [72]. Ductal elongation and secondary branching development are induced among others by the progesterone receptor type A (PR-A) [73] via increasing Msx2 expression [74]. A link between BMPs and PR-A involved in branching morphogenesis during postnatal mammary gland development has been reported [75]. In addition, BMPs are also involved in the myoepithelial compartmentalization and lumen formation [76]. Finally, an in vitro study using sorted undifferentiated mouse mammary epithelial cells demonstrated the importance of the BMP pathway in driving lactogenic differentiation [77].

All of these data demonstrate that BMP signaling constitutes an important regulator of the mammary gland during embryogenesis but most likely also during adulthood.

### 3.2. BMPs Are Key Embryonic Factors for Adult Tissue Modeling 

In healthy tissues, epithelial cells, as well as cells of the mammary gland environment (fibroblasts, adipose tissue cells, hematopoietic cells), contribute to the production of soluble BMP2 and BMP4 molecules [78]. These two cytokines regulate normal mammary epithelial cell functions. Indeed, SCs and progenitors sorted according to their CD10 and EPCAM expressions [79] were shown to express the different elements of the BMP pathway, indicating that in a physiological context, BMP molecules likely play a role in the regulation of breast SC. This was formally demonstrated by functional assay following exposure of different human cell types to soluble BMP2 or BMP4 [78] and further substantiated by the use of TGF/BMP inhibitors allowing the expansion of immature epithelial basal cells [80]. Interestingly, as shown in the hematopoietic system [81], BMP2 and BMP4 molecules have distinct effects on mammary SC regulation. Indeed, while BMP4 modulates MaSC and myoepithelial progenitors, BMP2 controls the commitment and proliferation of luminal progenitors [78]. This is consistent with results reported in the context of mammary gland development in mice showing that BMP2 was involved in the in vivo regulation of the luminal lineage [76]. Importantly, these results established that despite their close similarity, BMP2 and BMP4 exert different functions on the human mammary gland.

## 4. Early Events of Breast Carcinogenesis

### 4.1. BMP Signaling and Breast Cancer Initiation 

BMP signaling is also a well-known pathway that orchestrates the development and homeostasis of adult tissues such as the neural system [82] and is involved in the protection against neurotoxic compounds [83,84,85]. Studies on BMP signaling dysregulations in the neural system have been remarkably pioneering in the identification of their importance in cancer SC phenotype in glioblastoma [86,87]. Modulations of BMP signaling by epigenetic mechanisms such as methylation of BMP-receptor promoters have been of particular clinical interest to further stratify patients and propose new therapeutic strategies [86]. Since then, the importance of the BMP signaling alterations in cancer SC features has been extended to breast cancer and leukemia [78,88]. Indeed, our group unveiled that the microenvironment of human primary luminal breast tumors produces abnormally high amounts of soluble BMP2 compared to healthy tissue. This higher level is associated with BMPR1B overexpression by the tumor cells. These two observations argued in favor of a potential over-activation of the BMP pathway in the luminal tumor cells. To test this hypothesis, a model of mammary SCs (the MCF10A human, non-transformed epithelial SC cell line) was chronically exposed to high BMP2 concentrations. This drove MCF10A transformation towards a luminal tumor phenotype. Interestingly, addition of the IL6 pro-inflammatory cytokine further stabilized the transformed phenotype. The luminal phenotype of the tumors derives at least in part from a BMPR1B-initiated signaling cascade involved in luminal commitment of human mammary SCs in the normal context and leading to a FOXA1/FOXC1 transcription factor balance switch in favor of FOXA1, simultaneously with an up-regulation of GATA3 [78].

Surprisingly, BMP2-mediated luminal transformation of MCF10A is accompanied by a strong activation of the estrogen signaling pathway despite the absence of ERα66 in those cells. Our understanding of estrogen signaling is hindered by the existence of several isoforms generated by alternative splicing and different promoter usage [89]. These isoforms can be expressed in both ERα66-positive and -negative cells and display different sub-cellular localizations [90,91]. For example, unlike ERα66, ERα36 is expressed mainly at the plasma membrane and activates estrogen non-genomic signaling by activating the ERK pathway through an interplay with the MKP3 phosphatase [92]. Interestingly, in the context of EDC research, ERα36 displays altered ligand preference and causes distinct effects compared to ERα66. For instance, the tamoxifen drug used as an estrogen antagonist in ERα66 breast cancers behaves as an estrogen agonist for ERα36 [93,94]. The importance of these different ERα isoforms in mammary epithelial SC features and in the context of breast cancer has been confirmed by a recent study [94].

In addition, BMP signaling has also been directly implicated in thyroid-lineage specification [95,96] as well as in thyroid carcinoma [97]. Of note, thyroid hormone status interferes with estrogen target gene expression in breast cancer samples in menopausal women [98]. This highlights shared interests in further investigating the importance of the BMP pathway for both thyroid and estrogen signaling in the context of exposure to EDCs. Altogether, these different examples illustrate how BMP signaling, by controlling stem cells, is at the interface of a number of fundamental physiological processes in humans and is directly involved in mammary stem cell transformation.

### 4.2. Involvement of Bisphenols in Breast Carcinogenesis

During development and organogenesis, tissue differentiation is tightly regulated by a series of precisely timed molecular and cellular events. It is now widely accepted that adverse environmental exposure during these processes might have significant life-long implications [99,100,101]. Some of the first steps of carcinogenesis are an increase in proliferation, apoptosis inhibition, and activation of survival signaling pathways. To do this, several tumor suppressor genes, like p53 or Brca1 for instance, need to be inactivated by different mechanisms including epigenetic changes. 

Evidence gathered from studies in experimental models and human populations have already confirmed that EDCs, including BPA, contribute to increased disease risk [102,103]. Given the significant involvement of estrogens in both normal and pathological conditions, EDCs able to interfere with the homeostasis of the estrogen endocrine system are a potential source of several health disorders. A positive relationship between BPA exposure and cancer development is reported in the literature [104]. Although there are growing evidence that exposure to BPA may be harmful for human health, understanding of the molecular mechanisms underlying BPA-dependent effects in cancer development is still limited. Of note, one study reported that exposure of normal human mammary epithelial cells to BPA induces proliferation and senescence [105]. In contrast to non-exposed cells, these BPA-treated cells generate bigger mammospheres and are able to maintain their proliferation for a longer period of time due to autocrine growth factor secretion. The authors also observed an increase in DNA hypermethylation of tumor suppressor genes, such as Brca1. All these data support that BPA can promote early pre-tumoral stages as also shown in studies performed on normal human breast epithelial cells (MCF-10F) [106]. When mice were treated at puberty with BPA (added in their drinking water to mimic typical exposure in humans), estrogen-dependent transcriptional events were perturbed in their offspring and the number of terminal end buds was altered in a dose-dependent fashion [107]. Moreover, pubertal BPA exposure altered the function of MaSCs, leading to the appearance in the regenerated glands of early neoplastic lesions with molecular alterations similar to the ones detected in early neoplastic breast cancer tissues [108]. In addition, BPA-treated human mammary stem cell lines, such as MCF-10F cells, upregulate their expression of genes involved in DNA repair and downregulate pro-apoptotic gene expression [109]. Altogether, these observations strongly support that MaSCs are directly susceptible to BPA-induced transformation [107,108,109].

Mechanistically, estrogens and estrogen-mimetics like BPA were mainly thought to act through the canonical ERα66 receptor, a nuclear receptor that can initiate signaling pathways at the cell membrane and transcriptional responses in the nucleus. It was shown that BPA was capable of up-regulating the protein levels of steroid receptor coactivators (SRC-1, SRC-3), as well as promoting the activity of estrogen response elements [110]. Interestingly, it was demonstrated that in this case the addition of melatonin blocked the increase in steroid receptor coactivator expression and activity of estrogen response elements triggered by BPA [110]. Another study suggested that BPA stimulates the release of EGFR-ligands by directly targeting other molecules than ER, such as ADAM17 or ADAM10. A better understanding of BPA-specific effects on other molecules than ERα66 is much required to improve our knowledge of its tumorigenic potential [111].

### 4.3. A New Concept: Bisphenol Molecules as Breast Cancer Drivers through the Perturbation of Alternative Signaling Such as the BMP Pathway

**Figure 1:**
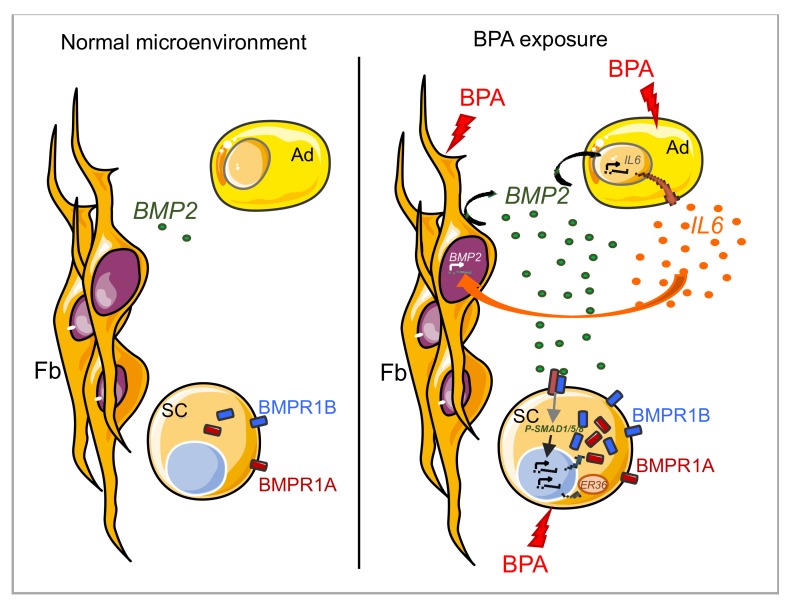
BPA-induced modifications of the mammary gland ecosystem. In the absence of BPA (**left panel**), BMP2 production by the breast microenvironment and BMP receptor expression by mammary epithelial cells sustains the homeostasis of the mammary gland. In the presence of BPA (**right panel**), BMP2 production by mammary fibroblasts is stimulated and in turn activates, in conjunction with BPA, IL6 production by adipocytes. IL6 can then further increase BMP2 production by fibroblasts. In parallel, BPA primes epithelial SCs cells to the increased BMP2 signaling by acting on BMP receptor expression and localization. This dysregulated BMP signaling and the inflammatory context provided by IL6 cooperate to favor mammary stem cell transformation.

Works from our team and others suggest that bisphenols could act on multiples cell types of the mammary gland, and its effects may converge to provoke major dysregulations of the BMP pathway that could participate in luminal breast cancer initiation. Indeed, we observed a major impact of BPA on the mammary microenvironment (niche) equilibrium. BPA greatly increases BMP2 production by stromal cells of the human mammary SC microenvironment [78]. In this context, we also demonstrated that long-term exposure (60 days) of human mammary stem cells to BPA initiates fundamental changes in stem cells, in particular by altering major BMP signaling elements such as receptor expression and localization [43]. This results in the “priming” of the SCs to exogenous activating molecules of the BMP pathway. Therefore, BPA sensitizes breast SCs to exogenous soluble BMP molecules. Collectively, these results suggest a possible model where non-genotoxic alterations in both the stem cells and their niche could act synergistically to initiate the transformation process (Figure 1). 

Interestingly, these previous studies showed that BPA impacts BMP signaling pathway members in both mammary epithelial and stromal cells that do not express ERα66. At the mechanistic level, it then remains to understand how BPA signals in these cells and especially if it is through other ERα isoforms or through ER-independent pathways. These questions are of great interest for understanding the effects of both BPA and estrogens since it has been reported that some cell lines are able to respond to an estrogen signal despite their very low levels or complete absence of estrogen receptors [99]. 

In response to accumulating evidence in favor of adverse health effects following exposure to BPA, likely mediated by its activation of ERα66, alternative bisphenols have been developed such as BPS and BPF that are considered safer due to their very low binding affinity to ERα [112]. However, an increasing number of studies are showing that these alternative bisphenol molecules are not as innocuous towards mammary gland biology and breast cancer as hoped [113]. We were indeed surprised to observe that BPA (high affinity binding to estrogen-receptors) as well as its substitute BPS (very weak affinity binding to estrogen-receptors) both induce BMP2 synthesis in the healthy breast stroma [78], raising the question whether these bisphenols mediate their transforming effects solely through a classical ER-dependent mechanism. Since then, other studies have shown that BPS, as well as BPF, induce similar effects as BPA [112,113]. Moreover, it was reported that BPA treatment increases aromatase expression and its activity in healthy breast fibroblasts, leading to an increase in estrogen biosynthesis and secretion. The same observations were made after treatment with BPS [114]. 

These results are particularly interesting with regards to the important role of the microenvironment in the different steps of carcinogenesis. Our work thus indicates that the BMP pathway could be altered by EDCs such as BPA and its proposed alternatives, both at the level of the stem cells and their microenvironment. This suggest that early detection of increased BMP2 levels in the mammary microenvironment may constitute a reliable marker of early transformation process and could be a valuable indicator of exposure to EDCs such as bisphenols.

## 5. Conclusions

Different signaling pathways often engage in complex interactions synergistically mediating an appropriate cellular response. Estrogen signaling is no exception and is likely involved in a crosstalk with the BMP pathway at multiple levels in the mammary gland. BMPs are secreted proteins active in a very large number of organs and tissues during development, adulthood and pathogenesis [115]. Previous work suggested a close interaction between ER− mediated estrogen signaling and the BMP pathway in different cell types of the mammary gland. In a model of mammary epithelial SCs (MCF10A cell line), E2 or known EDCs like BPA or BPS were able to potentialize SMAD activation by BMP2 [43]. 

Deciphering dysregulations of the BMP pathway has been remarkably useful in unveiling its importance in regulating cancer SC phenotypes in the neural system [86,87]. The role of BMP signaling alterations in sustaining cancer SC features has been extended by our own work in breast cancer and leukemia [78,88]. We have shown that chronic exposure to high concentrations of BMP2 drives the transformation of mammary SCs toward the luminal tumor subtype [78] through the binding to its BMPR1B receptor. We have also demonstrated that long-term exposure of human mammary SCs to pollutants such as BPA initiates fundamental changes in SCs by altering major BMP signaling components [43], thus “priming” the stem cells to exogenous BMP activation. In addition to this effect on epithelial stem cells we revealed an impact of BPA on the tumor microenvironment through the induction of the synthesis of high levels of BMP2 by normal fibroblasts and stromal cells [78].

This review focused on the early steps of the transformation. Tumor progression, metastasis, and resistance to treatments could also be regulated by bisphenols as briefly illustrated in the following chapters. Progression is generally characterized by elevated cell proliferation rates. Several studies have demonstrated the involvement of BPA in the proliferation of ER-positive as well as -negative cancer cells. However, it has also been shown that BPA can trigger proliferation via non-classical estrogen receptors, such as the estrogen-related receptor gamma (ERRγ) [116,117]. Several studies have demonstrated that BPS promotes breast cancer cell proliferation, notably through an ER-cyclin D1-CDK4/6-pRb-dependent pathway, but only in ER-positive breast cancer cells [118,119,120]. Moreover, it has also been reported that BPF has the same proliferative action as BPA, BPS, or estrogen treatment on ER-positive transformed cells, and as for BPS, this proliferative effect relies on cyclin D and E expression through ER-dependent pathways [118]. Similarly to BPA, BPS can also induce epigenetic and transcriptional changes in breast cancer cells, upregulating proliferation-, cellular attachment-, adhesion- and migration-related genes [121].

Metastasis has long been associated with late stages of cancer, but new hypotheses on the origin of metastasis have progressively emerged suggesting that it could be an inherent marker of tumor cells [122,123]. Metastasis dissemination is a dynamic process that involves several steps: Local invasion of cells from the primary tumor, intravasation leading to dissemination through the blood or lymph flux, extravasation to invade new tissues, implantation and finally new tumor growth. Numerous signaling pathways and programs are activated during this process, such as epithelial-to-mesenchymal transition (EMT), anoikis, migration and proliferation among others (for review: [124,125]). It has been shown that ER-negative breast cancers are associated with an increased risk of metastasis development [126]. Indeed, ER-negative breast cancers express more mesenchymal markers such as vimentin and N-cadherin or EMT-transcription factors that are required for metastasis initiation. Conversely, ER-positive tumors are associated with a more differentiated luminal phenotype, expressing epithelial markers (E-cadherin, ER, Foxa1 for instance). Accordingly, a down-regulation of the luminal-specific transcription factor Foxa1 was induced after BPA treatment in triple negative tumor cell lines leading to EMT induction and increased cell motility [127]. In this study, BPA treatment activated the PI3K/AKT pathway, leading to a down-regulation of epithelial genes associated with an up-regulation of mesenchymal genes. 

Resistance and relapse can be due to tumor adaptation or evolution. Indeed, therapies elicit a selective pressure on cells, which in turn develop resistance, notably by acquiring mutations. Tamoxifen resistance of ER-positive tumors can be caused by a loss of ER [128], its mutation or post-translational modification [129], among others. It has been shown that BPA is involved in chemoresistance [130] and notably in tamoxifen resistance in ER-positive tumor cell lines [131] by decreasing tamoxifen-induced apoptosis and increasing gene expression of ERRα, which contributes to tamoxifen resistance [132] and cell proliferation [117]. Another study has demonstrated that an ERα variant can be induced by BMP2 [133] and could be involved in tamoxifen resistance [134]. We can hypothesize that in ER-positive tumors, under tamoxifen treatment and in a BPA-containing environment, some cells acquire resistance to treatment through a switch in signaling pathways in favor of a decrease in treatment cytotoxicity and a modification of estrogen receptor stoichiometry, e.g., an increase in ERRα or ERα isoform expression. Treatment efficacy can then be bypassed and cells can start to proliferate again. 

**Figure 2:**
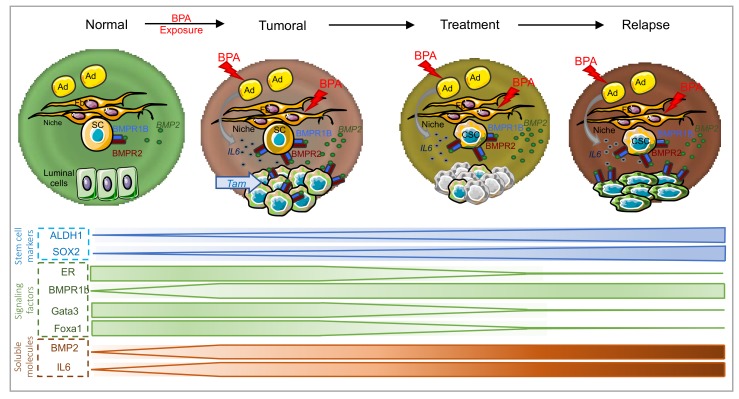
Putative mechanism of BPA involvement during mammary gland carcinogenesis. In the normal breast, the microenvironment secretes factors, BMP2 in particular, that regulate the luminal lineage [43]). Exposure to BPA induces dysregulations in the synthesis of microenvironmental factors: Mammary fibroblasts increase their BMP2 secretion [78] and adipocytes establish an inflammatory environment by releasing IL6 [135]. The microenvironment then becomes permissive to transformation, and in concert with BPA, leads to the initiation and progression of breast tumor. Treatments can kill the tumor bulk, but not cancer stem cells, leading to proliferation and relapse. The relapsing tumor has an aggressive phenotype due to its cancer stem cell origin and/or to pollutants that promote a decrease in epithelial marker expression (Gata3, Foxa1) [127], an increase in mesenchymal markers and stem-like features (ALDH1, SOX2) [136,137] or a loss of ER [128]. Fb, fibroblast; SC, stem cell; Ad, adipocyte; CSC, cancer stem cell; Tam, tamoxifen.

To conclude, the BMP signaling plays a major role in the regulation of SCs and of their microenvironment (niche), in both normal and tumor contexts. Multiple abnormalities of BMP signaling have been observed in cancer, but until recently had mostly focused on its role in advanced disease. However, due to the increasing documented importance of BMP signaling throughout breast cancer development (from initiation, progression, metastasis up to resistance), we suggest as a working model that BMP signaling alterations induced by exposure to BPA, such as increased levels of BMP2 and/or of BMP receptors activity, could participate to most stage of breast cancer development (Figure 2). This suggests that further investigating the alterations of the BMP pathway induced by exposure to bisphenols will improve our understanding of breast cancer etiology.
